# A case report of atypical anti-glomerular basement membrane nephritis associated with Mycobacterium Avium 

**DOI:** 10.5414/CNCS111254

**Published:** 2024-01-12

**Authors:** Julie Bech Jensen, Eva Gravesen, Sidse Graff Jensen, Iain Bressendorff

**Affiliations:** 1 Department of Nephrology,; 2 Department of Pathology, and; 3 Department of Medicine, Division of Respiratory Medicine, Herlev and Gentofte Hospital, Hellerup, and; 4 Department of Clinical Medicine, Faculty of Health and Medical Sciences, University of Copenhagen, Copenhagen, Denmark

**Keywords:** acute kidney injury, atypical anti-glomerular basement membrane disease, crescentic glomerulonephritis, non-tuberculous-mycobacteria, rapidly progressive glomerular nephritis

## Abstract

We present the case of a woman with atypical anti-glomerular basement membrane (anti-GBM) nephritis associated with concurrent pulmonary infection with *Mycobacterium avium*. A kidney biopsy showed crescentic glomerulonephritis with 50% active crescents and linear IgG staining, but no circulating anti-GBM antibodies were detected, and the patient did not have pulmonary hemorrhage. Despite treatment with a triple-regimen of antibiotics, corticosteroids, and plasmapheresis, the patient did not regain kidney function. One year later she is on maintenance dialysis and has still not cleared the infection with *M. avium*.

## Introduction 

Anti-glomerular basement membrane (anti-GBM) disease is a very rare form of small vessel vasculitis with rapidly progressive glomerular nephritis often concurrently associated with pulmonary hemorrhage with an estimated incidence rate of less than one per million per year [1]. An even rarer variant of anti-GBM disease is the atypical anti-GBM nephritis, which accounts for ~ 10% of cases with anti-GBM disease [2]. Atypical anti-GBM nephritis is characterized by linear IgG staining of the glomerular basement membrane on kidney biopsy, but negative anti-GBM antibodies in blood, absence of pulmonary involvement, and less rapid progression of kidney disease. 

We present a case with atypical anti-GBM nephritis associated with pulmonary infection with the non-tuberculous mycobacterium (NTM) *Mycobacterium avium*. 

## Case presentation 

A 64-year-old woman with chronic obstructive pulmonary disease and no history of kidney disease presented with 2 months of malaise, diarrhea, and a 10-kg weight-gain. She complained of progressive lower limb edema accompanied by severe dyspnea and a dry cough, but no arthralgias or rash. She had self-isolated for several months prior to presentation due to the SARS-CoV-2 pandemic. The patient was a current smoker and had no family history of kidney disease or vasculitis. Her father had died from tuberculosis when she was a child. 

On examination she was pale with pitting edema of both lower extremities and shortness of breath. She had blisters on her eyelids and lips. Blood pressure was 176/79 mmHg, and oxygen saturation was 98% on room air. She did not have a fever. 

## Results 

Blood tests revealed a creatinine of 433 μmol/L, hemoglobin of 6.6 mmol/L, total leucocytes 4.2 × 10^9^/L with neutrophilic dominance, platelets 124 ×10^9^/L, C-reactive protein of 44 mg/L, and albumin of 25 g/L. One month prior to presentation, plasma creatinine was 119 μmol/L. Urine was positive for albumin and hemoglobin by dipstick, and 24-hour urine collections showed protein excretion of 1.1 – 3.2 g on 5 consecutive days. The patient was not oliguric. 

Serological markers were normal or negative on repeated testing for anti-GBM antibodies, myeloperoxidase, and proteinase-3 antineutrophil cytoplasmic antibodies (ANCA), antinuclear antibodies, C3 and C4, cryoglobulin, rheumatoid factor, HIV, free light chain-ratio, immunoglobulins, and M-spike. 

Erythrocyte mean corpuscular volume and erythrocyte distribution width were both normal, transferrin saturation was 14%, and ferritin was 455 μg/L. 

**Figure 1:**
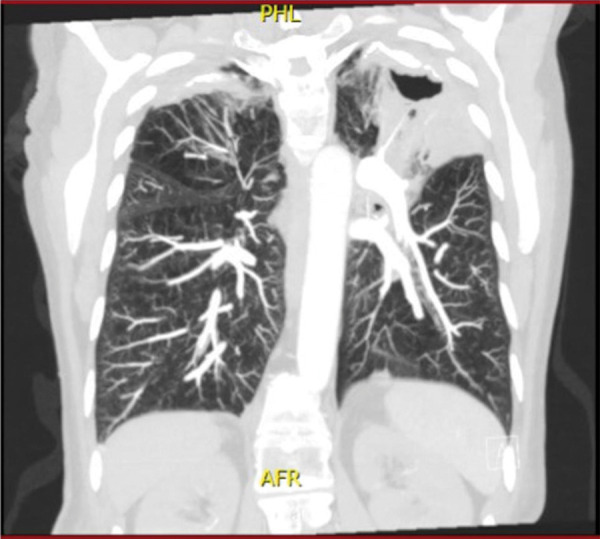
Computed tomography scan of the lungs revealing emphysema, bilateral infiltration, and calcification located in the upper lobes, and a cavity with no air-fluid level on the upper left lobe. There are no signs of pulmonary hemorrhage.

Computed tomography scan revealed emphysema, bilateral infiltration and calcification located in the upper lobes of the lungs but no signs of pulmonary hemorrhage, and a cavity with no air-fluid level on the upper left lobe (Figure 1). Both kidneys were of normal shape and size. 

**Figure 2:**
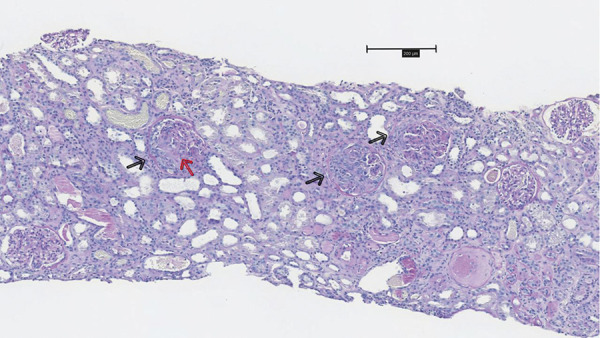
A kidney biopsy showing crescentic (black arrow) glomerulonephritis involving 50% of glomeruli with focal and segmental fibrinoid necrosis (red arrow) as well as focal and segmental endocapillary proliferation in 50% of the remaining glomeruli. There is minimal fibrosis and tubular atrophy, normal arteries, and no granulomas.

**Figure 3:**
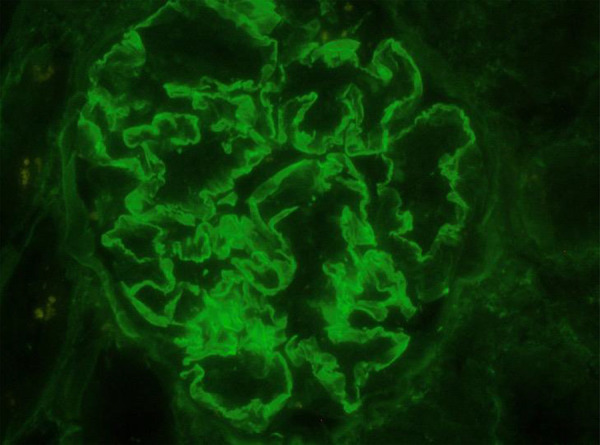
Immunofluorescence shows linear staining for IgG 1 – 2+ along capillary walls.

A kidney biopsy revealed crescentic glomerulonephritis involving 50% of glomeruli (16/32) with focal and segmental fibrinoid necrosis as well as focal and segmental endocapillary proliferation in 50% of the remaining glomeruli (Figure 2). Three (10%) glomeruli were globally sclerosed, and there was minimal fibrosis and tubular atrophy, patent capillary loops, normal arteries, and no granulomas. Immunofluorescence revealed linear staining for IgG 1 – 2+ along capillary walls (Figure 3) with co-dominant staining for κ and λ (both 3+) and focal staining for C3. There was no deposition of IgA, IgM, or C1q. We did not stain for IgG subclasses, and tissue was not sent for electron microscopy. 

Due to the presence of a pulmonary cavity and a family history of tuberculosis, we suspected a pulmonary infection with *M. tuberculosis*. Sputum cultures contained numerous acid-resistant rods, which were identified as *M. avium* by polymerase chain reaction (PCR). Blood cultures grew no bacteria, tests for SARS-CoV-2 were negative by PCR, plasma galactomannan was normal, and transesophageal echocardiography showed no vegetations on the heart valves. 

The clinical and histological presentation was consistent with atypical anti-GBM nephritis, which was considered to be caused by the pulmonary *M. avium* infection. Treatment was initiated with methylprednisolone pulses of 500 mg per day for 3 days followed by oral prednisolone 60 mg/day, and plasmapheresis every other day for a total of 14 sessions, but not hemodialysis. Cyclophosphamide was withheld due to the risk of exacerbating the pulmonary infection. Treatment of the mycobacterial infection was initiated with rifampicin, moxifloxacin, and azithromycin. 

The patient developed progressive thrombocytopenia after initiation of treatment with corticosteroids and plasmapheresis. To rule out bone marrow infiltration by *M. avium*, a bone marrow biopsy was performed, which showed a hypocellular marrow with normal megakaryocytes, but no granulomas or mycobacterial infiltration. The thrombocytopenia resolved after cessation of plasmapheresis and was subsequently attributed to membrane adsorption of thrombocytes during plasmapheresis. 

Unfortunately, the patient was unable to tolerate the antibiotic treatment due to side effects, and treatment was discontinued after 15 weeks. The patient did not recover kidney function, and hemodialysis was initiated after 1 month. One year later, the patient is still on dialysis and has not cleared the *M. avium* infection despite reinitiating antibiotic treatment, which has been tolerated for 9 months so far. 

## Discussion 

Rapidly progressive glomerulonephritis related to NTM is very rare. We were only able to retrieve two prior case reports of rapidly progressive glomerulonephritis in relation to NTM – one with ANCA-associated vasculitis [3] and one with double-positive ANCA and anti-GBM [4] disease. To the best of our knowledge, this is the first case report of atypical anti-GBM nephritis associated with NTM. Atypical anti-GBM nephritis has been described in association with other infectious agents (for example *Staphylococcus aureus* [5], bacterial pneumonia [6], parainfluenza [7], and possibly also cytomegalovirus [8]). In the largest published cohort of atypical anti-GBM nephritis, ~ 13% of cases were associated with a prodromal infection [9]. Given the overlap in symptoms with atypical anti-GBM nephritis and the difficulty in diagnosing some less virulent infections, it is possible that the incidence of prodromal or concurrent infections is even higher. 

In retrospect, treatment with corticosteroids and plasmapheresis may have delayed the time until clearance of the M. avium infection. It is of interest to note that in the large cohort of atypical anti-GBM nephritis [9] kidney outcomes were independent of treatment with immunosuppression. We speculate that the clinical outcome may have been better had we not used immunosuppressive therapy, potentially allowing for faster clearance of the presumed initiating and perpetuating infectious agent. In one of the previously reported cases of infection-related atypical anti-GBM nephritis (suspected *S. aureus* (not confirmed by culture or PCR) and no biopsy of lung or kidney tissue) [5], clinical remission was achieved after 8 weeks of antibiotic treatment without the use of immunosuppressive therapy. In the remaining cases of atypical anti-GBM nephritis associated with bacterial pneumonia [6], parainfluenza [7], and cytomegalovirus [8], kidney function declined despite concomitant treatment with antibiotics/antivirals, immunosuppression, and plasmapheresis. However, given the slow replication rate of NTM and long duration of antibiotic treatment necessary to treat the infection (12 months after culture conversion [10]), it is possible that regardless of concurrent immunosuppressive treatment, the infection in our case report could not be cleared fast enough to allow for immunological quiescence and recovery of kidney function. 

Given the extreme rarity of this condition, firm treatment recommendations are unlikely ever to become available. However, based on the limited available evidence, immunosuppressive therapy did not appear to be effective in the cases of infection-related atypical anti-GBM nephritis that we retrieved. 

In conclusion, atypical anti-GBM nephritis is a very rare condition, which in some cases may be triggered by an infectious agent. Outcomes are generally better as compared to anti-GBM disease, and kidney function may improve without the use of immunosuppressive therapy if the infectious agent can be successfully treated. 

## Informed consent 

The patient provided written informed consent to the publication of this case report. 

## Funding 

The authors received no financial support for the research, authorship, and/or publication of this article. 

## Conflict of interest 

JBJ, EG, and SGJ declare no conflict of interest. IB has received lecturing fees from Amgen and Bayer.
